# Complex Management of Bilateral Congenital Hydronephrosis in a Pediatric Patient: A Multidisciplinary Approach

**DOI:** 10.3390/healthcare13090998

**Published:** 2025-04-25

**Authors:** Nadica Motofelea, Ionela Florica Tamasan, Sonia Aniela Tanasescu, Teodora Hoinoiu, Jabri Tabrizi Madalina Ioana, Gheorghe Nicusor Pop, Alexandru Catalin Motofelea

**Affiliations:** 1 Doctoral School, “Victor Babes” University of Medicine and Pharmacy Timisoara, 300041 Timisoara, Romania; nadica.motofelea@umft.ro (N.M.); madalina.plic@umft.ro (J.T.M.I.); alexandru.motofelea@umft.ro (A.C.M.); 2 Department of Obstetrics and Gynecology, “Victor Babes” University of Medicine and Pharmacy Timisoara, Eftimie Murgu Sq. No. 2, 300041 Timisoara, Romania; 3 Department XI: Pediatrics, “Victor Babes” University of Medicine and Pharmacy Timisoara, 300041 Timisoara, Romania; tanasescu.sonia@umft.ro; 4 Center for Advanced Research in Cardiovascular Pathology and Hemostaseology, “Victor Babes” University of Medicine and Pharmacy Timisoara, 300041 Timisoara, Romania; tstoichitoiu@umft.ro; 5 Department of Clinical Practical Skills, “Victor Babes” University of Medicine and Pharmacy Timisoara, 300041 Timisoara, Romania; 6 Center for Modeling Biological Systems and Data Analysis (CMSBAD), “Victor Babes” University of Medicine and Pharmacy Timisoara, 300041 Timisoara, Romania; pop.nicusor@umft.ro; 7 Center of Molecular Research in Nephrology and Vascular Disease, “Victor Babes” University of Medicine and Pharmacy Timisoara, Eftimie Murgu Square No. 2, 300041 Timisoara, Romania

**Keywords:** congenital hydronephrosis, urinary tract infections, multidisciplinary approach, recurrent urinary tract infections, neurogenic bladder

## Abstract

Congenital anomalies of the kidney and urinary tract (CAKUT) are common developmental malformations and a leading cause of pediatric renal dysfunction. Severe hydronephrosis, especially when accompanied by ureteral duplication, ureterocele, or neurogenic bladder, poses significant diagnostic and therapeutic challenges. This case report presents a 7-year-old male with prenatally diagnosed bilateral grade IV/V hydronephrosis (according to the radiology hydronephrosis grading system), complicated by the right pyeloureteral duplication, the left ureterocele, and the neurogenic bladder. The patient’s clinical course was marked by recurrent urinary tract infections (UTIs), progressive renal dysfunction, and multiple surgical interventions. Initial decompression via bilateral ureterostomy and stenting led to significant improvements in renal function. However, the patient experienced recurrent febrile UTIs caused by multidrug-resistant pathogens, necessitating repeated hospitalizations and intravenous antibiotic therapy. Serial imaging studies documented persistent hydronephrosis, a neurogenic bladder, and vesicoureteral reflux. Subsequent surgical interventions included bilateral ureteral reimplantation, excision of the left ureterocele, and removal of a fibroepithelial polyp from the bladder wall. Despite these interventions, residual left hydronephrosis and right kidney hypoplasia persisted, underscoring the need for long-term surveillance. This case highlights the diagnostic and therapeutic challenges of managing CAKUT and emphasizes the importance of a multidisciplinary approach integrating imaging, functional assessment, and surgical planning. Early diagnosis and timely intervention can stabilize renal function, but ongoing monitoring and individualized treatment remain crucial for optimizing long-term outcomes in children with complex CAKUT.

## 1. Introduction

Congenital anomalies of the kidney and urinary tract (CAKUT) are among the most frequently detected prenatal malformations, affecting up to 1–5% of pregnancies and accounting for 20–30% of all anomalies identified antenatally [1,2]. For a fetus diagnosed with severe bilateral hydronephrosis at 30 weeks of gestation, the journey from prenatal ultrasound to long-term management exemplifies the profound challenges these conditions pose. With a prevalence ranging from 0.3 to 1.6 per 1000 live and stillborn newborns, CAKUT represents not only a common developmental anomaly but also the leading cause of pediatric end-stage kidney disease [1,3,4,5]. Neonatal hydronephrosis can result from various issues, including ureteropelvic junction obstruction (UPJO), ureterovesical junction obstruction, megacystis, megaureter, and vesicoureteral reflux (VUR) [6].

Among these, congenital hydronephrosis is defined as the dilation of the renal pelvis and calyces due to impaired urine flow, which is the most prevalent, detectable in 0.3–5% of prenatal ultrasounds [3,4,7]. While two-thirds of cases resolve spontaneously, the presence of associated anomalies such as ureteral duplication, ureterocele, and neurogenic bladder markedly increases complexity and risk [8].

The advent of maternal ultrasonography in the 1980s revolutionized the detection of antenatal hydronephrosis (AHN), yet it introduced a diagnostic paradox: early identification can mitigate renal damage from obstruction and infection, but it also risks overdiagnosis of transient conditions [2]. Severe congenital hydronephrosis, particularly grades IV/V, presents a unique challenge due to its unpredictable progression. The wide variation in clinical outcomes makes it challenging to distinguish between significant obstructions that necessitate surgery and those that might resolve on their own without intervention [9]. The unpredictability arises from the fetal kidney’s unique response to obstruction, which differs from postnatal physiology, and is further complicated by the absence of reliable predictive tools [9]. Current management relies on ultrasonography, renal pelvis anterior–posterior diameter, and nuclear medicine assessments, yet these fall short in identifying surgical candidates with precision [7]. Emerging evidence points to genomic imbalances as a contributor to CAKUT pathogenesis, suggesting a need for genetic screening, but noninvasive biomarkers for renal deterioration remain elusive [10,11].

These diagnostic and prognostic uncertainties underscore the necessity of a multidisciplinary approach to pediatric hydronephrosis. Collaborative care integrating urologists, nephrologists, and surgeons has shown promise across medical fields—improving functional outcomes in diabetes and reducing mortality in acute care settings—yet its impact on survival in CAKUT remains understudied [12,13,14]. In complex cases, such coordination enhances decision-making, disease control, and service delivery, addressing the multifaceted needs of patients with severe anomalies [15]. However, gaps persist in optimizing this approach, particularly in tailoring interventions to prevent long-term renal impairment while avoiding unnecessary procedures [16].

This case report examines a 7-year-old male with prenatally diagnosed bilateral grade IV/V hydronephrosis, complicated by right pyeloureteral duplication, a left ureterocele, and a neurogenic bladder. His clinical course, marked by recurrent urinary tract infections (UTIs), progressive renal dysfunction, and multiple surgical interventions, exemplifies the diagnostic and therapeutic challenges of severe CAKUT. Through a detailed analysis of his journey from prenatal detection to stabilization at age 7, this study aimed to unravel the complexities of managing such cases, assess the effectiveness of multidisciplinary care, and underscore lessons for enhancing treatment strategies. By exploring these challenges within the framework of the existing literature, this report aspires to contribute to the development of more precise diagnostic and management frameworks for pediatric urological anomalies.

## 2. Case Presentation

A 7-year-old male was diagnosed prenatally at 30 weeks of gestation with bilateral congenital hydronephrosis, classified as grade IV/V (according to the radiology hydronephrosis grading system) [17]. Postnatal imaging confirmed significant bilateral hydronephrosis grade (IV/V), pyeloureteral duplication on the right side, and a left ureterocele at the vesicoureteral junction. The patient’s clinical course and management were marked by multiple complications, including recurrent urinary tract infections (UTIs), progressive renal dysfunction, and repeated surgical interventions.

### 2.1. Initial Presentation and Neonatal Period

This is the case of a 7-year-old child born in 12 February 2018 and diagnosed prenatally at 30 weeks of gestation with bilateral congenital hydronephrosis, grade IV/V. The patient was born at 38 weeks of gestation via a cesarean section with a birth weight of 3200 g. Apgar scores were 8 and 9 at 1 and 5 min. On the third day of life, the patient was transferred from the Neonatology Department to the Pediatric Surgery Department for the management of hydronephrosis with the following diagnoses: bilateral grade IV/V hydronephrosis, right pyeloureteral duplication, and a left ureterocele at the vesicoureteral junction.

**Figure 1:**
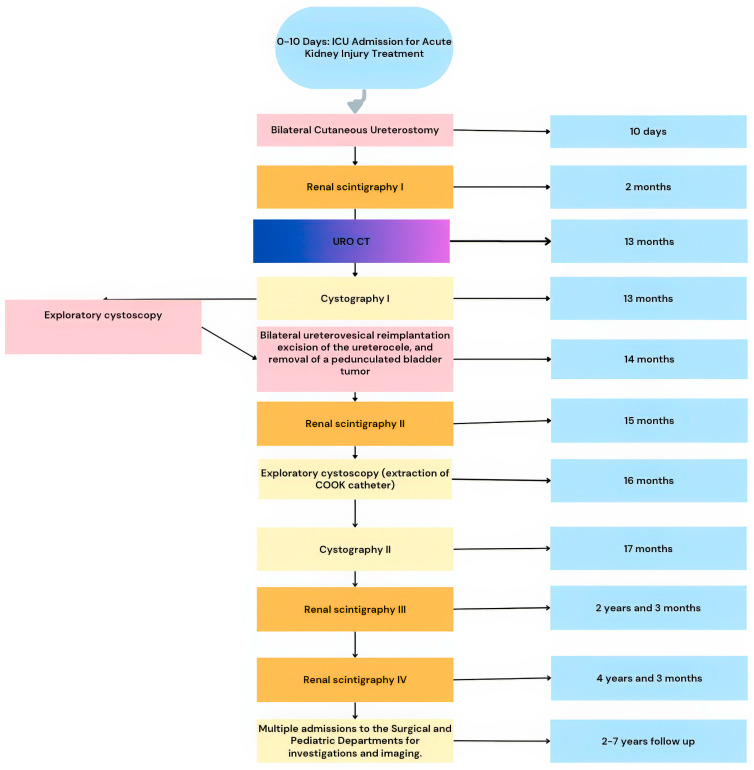
Flowchart of case management.

Within the first 12 h after admission to the Pediatric Surgery Department, the patient’s general condition worsened, prompting a transfer to the Intensive Care Unit. A 6Fr Foley catheter was inserted, and clinical and laboratory monitoring continued. The newborn presented with neonatal icteric syndrome. Following a deterioration in condition and the insertion of a Foley catheter, transient macroscopic hematuria was observed. In the subsequent hours, serum creatinine levels increased, and oliguria appeared. The general condition continued to deteriorate without identification of the pathogenic agent. In the meantime, fever developed, along with mottled skin and a progressive increase in serum creatinine. Empirical antibiotic therapy was initiated, along with pathogenic and symptomatic treatment. The evolution was slowly favorable until day 10 of life when surgical intervention could be performed. The entire case management is presented in Figure 1.

Surgical Interventions and Recurrent Complications:

At three months of age, the patient underwent bilateral cutaneous ureterostomy and stenting to relieve obstruction. Despite this intervention, he experienced recurrent febrile UTIs caused by *Klebsiella pneumoniae*, *Escherichia coli*, and *Pseudomonas aeruginosa*, necessitating repeated hospitalizations and intravenous antibiotic therapy. A notable episode at nine months involved a *Klebsiella pneumoniae* UTI, which was initially treated orally but required escalation to dual intravenous antibiotics due to an allergic reaction. The patient suffered from one of the most severe urinary tract infections. Additionally, they had a history of previous infections that were managed at home with oral antibiotics based on urine culture results.

### 2.2. Subsequent Imaging and Interventions

Serial imaging studies, including uro-CT, ultrasonography, and voiding cystourethrography (VCUG), documented persistent hydronephrosis, a neurogenic bladder, and evidence of vesicoureteral reflux (VUR) on the left side. Renal ultrasonography revealed right kidney hypoplasia with a bifid renal pelvis and left kidney compensatory hypertrophy with grade III/IV hydronephrosis. Renal scintigraphy demonstrated differential renal function of 86% for the left kidney and 14% for the right kidney. VCUG findings showed a neurogenic bladder with diverticula and passive left VUR. At 14 months of life, the patient underwent bilateral ureteral reimplantation, excision of the left ureterocele, and removal of a fibro-epithelial polyp from the bladder wall. The fibro-epithelial polyp resulted from the proliferation of bladder mucosa, which occurred because the bladder was excluded from function for 13 months. Postoperative recovery was complicated by transient hematuria and dysuria, but renal function stabilized.

**Figure 2:**
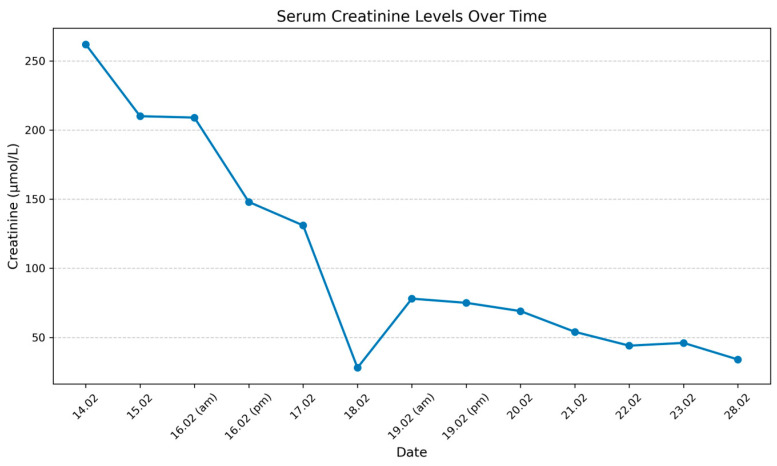
Serum creatinine levels.

The graph shows the evolution of serum creatinine levels in a pediatric patient during hospitalization in the Department of Pediatric Surgery. Initially, on 14 February (the 3rd day of life), creatinine was elevated at 262 μmol/L, reflecting significant renal impairment. Over the next few days, creatinine levels steadily decreased, reaching 131 μmol/L on 17 February, indicating an improvement in renal function. By February 18th, there was a sharp decline to 28 μmol/L, suggesting a marked recovery in renal clearance (Figure 2).

Between 19 February and 22 February, creatinine levels showed minor fluctuations, with a slight increase to 78 μmol/L on 19 February before stabilizing and continuing to decline, reaching 44 μmol/L on 22 February, coinciding with surgical intervention (Figure 2). Postoperatively, creatinine levels remained stable and further decreased to 34 μmol/L by 28 February, reflecting sustained improvement in renal function (Figure 2).

Throughout the hospitalization, no pathogens were isolated in urine cultures.

On the 10th day of the newborn’s life, after completing the preoperative preparations, the surgical procedure was carried out, which included the following:

- Bilateral cutaneous ureterostomy (2 stomas on the right, 1 stoma on the left, with complete exclusion of bladder function),
- Bilateral ureteral stenting.

Due to the ureterovesical obstruction caused by the left ureterocele, there were no indications for percutaneous nephrostomy, but rather for cutaneous ureterostomy, as the obstruction was located distally due to the left ureterocele at the vesicoureteral junction.

After the intervention was performed, the patient was transferred to the Neonatology Clinic for clinical and laboratory monitoring, as well as for completing post-operative treatment and investigations where he would remain hospitalized for 13 days. At admission, in the neonatology department, the general condition was moderately impaired, with axial hypotonia, marked psychomotor agitation, pale-mottled skin, and a capricious appetite.

During hospitalization, the infant’s condition steadily improved, with clinical stabilization, enhanced laboratory parameters, and gradual weight gain. Upon discharge, the toddler was in good overall condition. Moreover, the patient was afebrile, had an appetite, and presented diuresis via functional ureterostomy. During the hospitalization, the medial treatment included antibiotics (Meropenem and injectable Amikacin), antipyretics (Perfalgan solution), vitamin D supplementation (Vigantol), hydro-electrolytic support, and careful clinical and laboratory monitoring.

The patient experienced multiple episodes of urinary tract infections (UTIs) requiring repeated hospitalizations for investigations and treatment during the first year of life.

At 4 months of age, the infant was readmitted in the Pediatric Surgery Department for the treatment of an active UTI (urinary tract infection) and monitoring of bilateral ureterostomy associated with CAKUT.

These episodes underscore the importance of continuous monitoring for patients with a history of congenital hydronephrosis and ureterostomy, given the high risk of recurrent UTIs, which can negatively affect long-term renal function.

Consequently, the toddler remained with a bilateral cutaneous ureterostomy until reaching 14 months of age.

### 2.3. Significant Infections

Following the cutaneous ureterostomy, the child experienced multiple infections. These were diagnosed both after the onset of urinary tract infection symptoms and during routine evaluations of ureterostomy cultures. Most of these infections were managed with oral antibiotics.

At nine months of life, the infant presented a urinary tract infection caused by *Klebsiella pneumoniae*. Oral treatment was attempted, but the infant developed an urticarial-type allergic skin reaction on the lower limbs, leading to the initiation of double injectable antibiotic therapy with Cefotaxime (0.8 g/day) and Gentamicin (0.05 g/day). This was one of the most severe infections, complicated by urosepsis.

Subsequently, at ten months of age, the infant was diagnosed with acute pyelonephritis caused by *Escherichia coli*, as confirmed by urine culture. This hospitalization was crucial for managing a severe urinary infection and stabilizing the overall condition; the treatment involved injectable Amikacin (128 mg/day) for ten days.

At one year of age, another hospitalization became necessary due to a urinary tract infection caused by *Pseudomonas aeruginosa* and *Staphylococcus aureus*, as confirmed by urine culture. This episode underscored the risk of recurrent infections in the context of renal malformation, and treatment with Gentamicin (60 mg/day) was administered. The final documented infection involved *Enterococcus*.

Later, at thirteen months of age, extended hospitalization was required for clinical and imaging evaluation and monitoring, particularly given the context of recurrent urinary tract infections and the resolution of the bilateral ureterostomy (see Table 1).

In addition to these infections requiring hospitalization and injectable antibiotic treatment, the child experienced numerous recurrent urinary tract infections that were managed on an outpatient basis with oral antibiotics.

Table 1 illustrates the presence of multidrug-resistant microorganisms responsible for the urinary tract infections in the child and underscores the importance of administering broad-spectrum antibiotics.

### 2.4. Imaging

URO-tomography was performed at 13 months of life.

Conclusions: Bilateral ureterostomy, right renal hypoplasia with pyelocalyceal duplication.

Findings: Postoperative status with bilateral ureterostomy; distal ureteral ends expressed subcutaneously in the suprapubic region.

Right kidney: Reduced size, pyelocalyceal duplication, bifid renal pelvis; upper pelvis dimensions 20/10 mm, parenchyma 3.5 mm, delayed secretion and excretion; lower pelvis dimensions 28/22 mm, parenchyma 6 mm, secretion and excretion within physiological limits. Ureter with a normal diameter and homogeneous opacification.

Left kidney: Size 72/31 mm, parenchyma 9.5 mm, secretion and excretion within physiological limits, no pyelocalyceal dilatation or radio-opaque calculi.

Bladder: Empty, without contrast retention or mural lesions.

Normal findings for the liver, pancreas, gallbladder, adrenal glands, and basal lungs.

**Figure 3:**
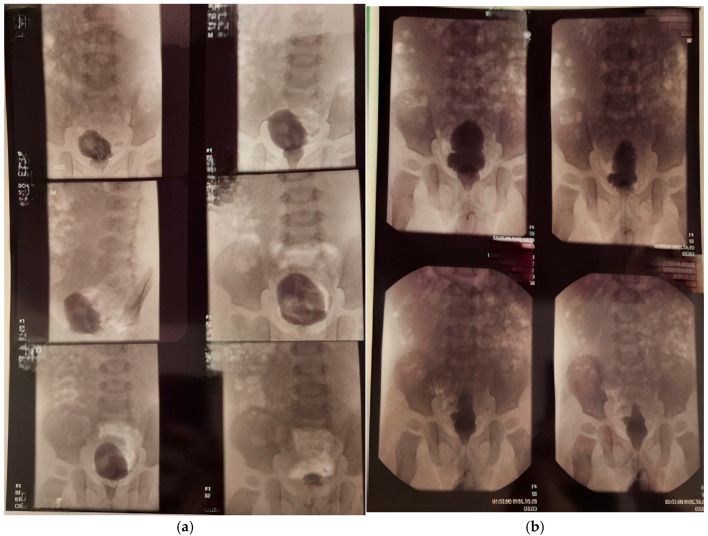
Cistography evaluation at two time points. (**a**) At 1 month and 20 days (**b**) At 1 year, 4 months, and 25 days (2 months post-surgery).

The first cystography was performed at 1 month and 20 days of life (Figure 3a, imaging on the left side). Findings: bilateral ureterostomy. Bladder in normal position, reduced in size, with smooth but slightly lobulated contours, and homogeneously opacified. Passive and active vesicoureteral reflux on the left side.

At 1 year, 4 months, and 25 days of life, voiding cystourethrography was performed at 2 months distances from the surgery. The second cystography was showing an irregular, pseudo-diverticular contour of the urinary bladder and a thickened bladder neck. No vesicoureteral reflux or post-void residual urine was identified (Figure 3b, imaging on the right side).

At the reevaluation by pediatric nephrology, ultrasonography revealed that the right kidney was normally located with a longitudinal axis of 5 cm, an irregular contour, relatively blurred corticomedullary differentiation, and a moderately hyperechoic appearance. The renal pelvis had an anteroposterior diameter of 0.4 cm without evidence of calculi. The left kidney was also normally located with a longitudinal axis of approximately 7.6 cm, displaying slight pyelocaliceal hypotonia without calculi. The urinary bladder was empty with thickened walls, and the ureters were not visualized. Bilateral ureterostomy was present.

At 14 months of age, the patient underwent a second surgical intervention beginning with exploratory cystoscopy. The subsequent surgical steps included bilateral ureterovesical reimplantation, excision of the ureterocele, and removal of a pedunculated bladder tumor. Histopathological examination revealed the tumor to be a fibroepithelial bladder polyp.

Intraoperatively, the urethra was found to have a normal caliber with no posterior urethral valves. The bladder mucosa displayed multiple diverticula, characteristic of a “fighting bladder”. The right kidney exhibited pyeloureteral duplication. The ureteral orifice of the lower renal pelvis was in a normal position and associated with a ureterocele, while the orifice of the upper renal pelvis was located inferior to the verumontanum. A pedunculated tumor, approximately 1 cm in diameter, was identified near the right ureteral orifice. The left ureteral orifice was enlarged, resembling a “golf-hole”. Ureteral stumps were explored, with the right ureter examined up to 0.5 cm and the left ureter up to 1 cm.

The surgical procedure involved a Pfannenstiel incision followed by the dissection of subcutaneous tissue and a longitudinal cystostomy. The pedunculated tumor, with a yellowish-red color and firm consistency, was excised and sent for histopathological examination. The ureterocele and the distal end of the left ureter were removed. The left ureterostomy site was circumferentially incised, and the left ureter, of normal caliber, was dissected proximally and distally over approximately 5 cm. Bilateral ureterovesical reimplantation was successfully performed. Catheters were then placed, including a COOK 4Fr/14 cm catheter on the upper right renal pelvis, a 3.5Fr/14 cm catheter on the lower left ureter fixed to the bladder mucosa, and a Foley bladder catheter (10Fr). The bladder was repaired in two layers, and a prevesical drain was placed and exteriorized laterally to the bladder on the right side. The abdominal wall layers and skin were sutured anatomically.

The histopathological examination confirmed a fibroepithelial polyp of the bladder mucosa.

Post-surgical abdominal ultrasound performed at 15 months of age revealed that the right kidney measured 51 × 30 mm, with a parenchymal thickness of 5 mm, a renal pelvis of 15 mm, and calyces measuring 9 mm. The left kidney measured 81 × 31 mm, with a parenchymal thickness of 7–8 mm, a renal pelvis of 15 mm, and calyces measuring 5 mm. The Cook catheter was correctly positioned. The right kidney showed pyelocalyceal duplication, with the lower unit not displaying the Cook catheter, while the upper unit had reduced dimensions and parenchyma and was difficult to visualize; the Cook catheter was present in this region. The liver, spleen, bile ducts, pancreas, and urinary bladder appeared normal, and peritoneal recesses were free of fluid.

**Figure 4:**
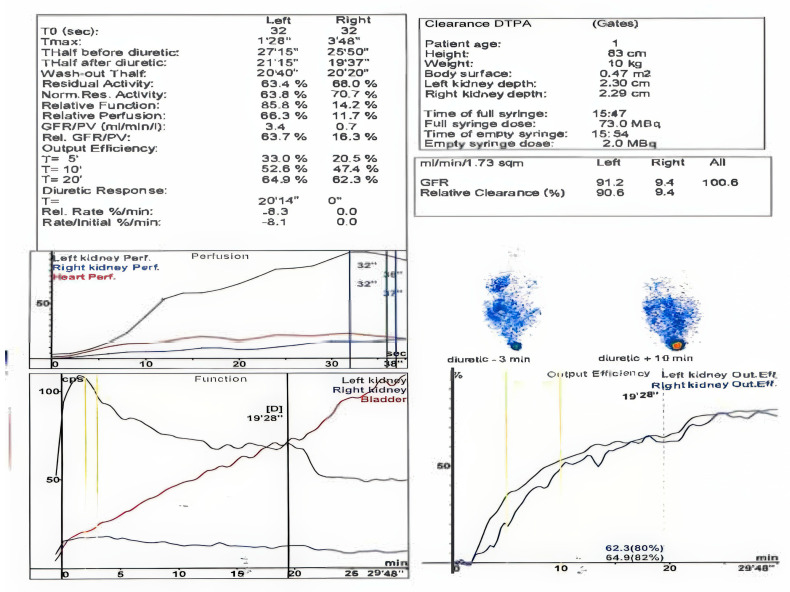
Renal scintigraphy at two months of life.

At 18 months of life, the child was rehospitalized for surgical reevaluation. Under general anesthesia, exploratory cystoscopy (Figure 3b) revealed that the bladder mucosa exhibited a “fighting bladder” appearance with pseudopolypoid and pseudodiverticular changes. The distal ends of the Cook catheters were observed at the ureteral orifices. The Cook catheters were removed with favorable postoperative evolution (Figure 4).

Over the course of these seven years, four renal scintigraphies were conducted. The first procedure took place when the child was just two months old. The first renal scintigraphy was performed using 99M TC–DTPA. The results indicated that the left kidney was in a normal position, slightly hypertrophic, with irregular contours, normal perfusion, and homogenous parenchymal uptake. The glomerular filtration rate (GFR) was 91.2 mL/min. Spontaneous excretion was observed, with transient lower ureteral dilation but no evidence of stasis in the excretory pathways by the end of the study. The renogram showed a normal peak at 2 min with a descending excretory slope, slightly elevated T1/2, and activity retention (AR). A non-obstructive response to furosemide was noted.

The right kidney was in a normal position but reduced in size, showing reduced perfusion with delayed peak uptake, heterogeneous parenchymal distribution, and a GFR of 9.4 mL/min. Excretion was spontaneous but reduced and slow, without evidence of obstruction or stasis in the excretory pathways by the end of the study. The renogram revealed a flattened peak with reduced amplitude at 4 min and a slowly ascending excretory segment with delayed T1/2 and increased residual activity at 20 min. A non-obstructive response to furosemide was also observed.

**Figure 5:**
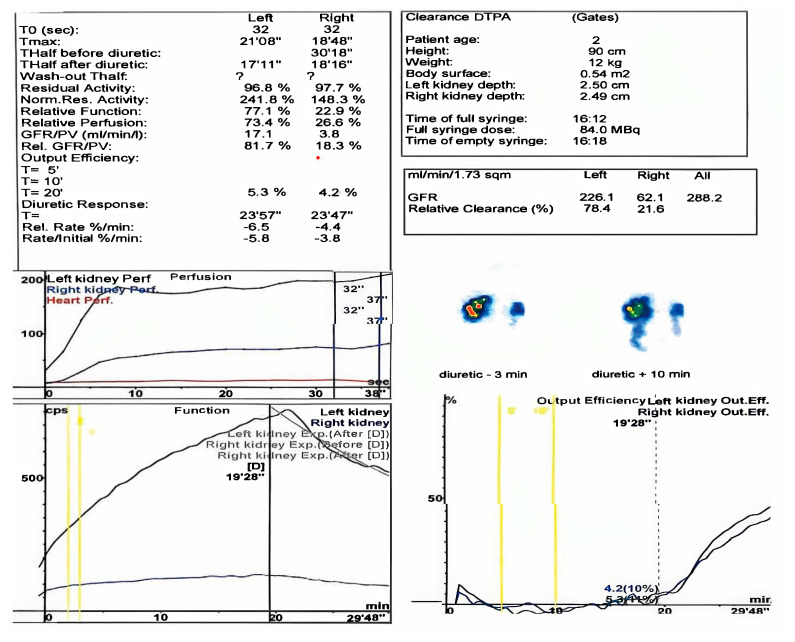
The second renal scintigraphy.

The differential functional contribution was 86% for the left kidney and 14% for the right kidney, with a total GFR of 100.6 mL/min. The conclusion was that the right kidney was hypoplastic with significantly diminished function (Figure 5).

When the second renal scintigraphy was conducted, the child was 15 months old, occurring one month after the second surgical procedure (ureterocystoplasty with tumor excision and bilateral ureteral reimplantation in the context of duplex system and ureterocele). The second renal scintigraphy revealed that the left kidney was enlarged, compensatorily hypertrophic, with regular contours, normal perfusion, and homogeneous parenchymal uptake. The GFR was 226 mL/min. Delayed excretion was noted, likely due to insufficient hydration, with post-diuretic improvement. Persistent pyelocaliceal and ureteral stasis were observed throughout the study, with a tortuous ureter in the mid-third. The renogram showed delayed peak uptake and an ascending excretory segment until diuretic administration. Post-diuretic normalization of T1/2 was observed, though stasis persisted in the excretory pathways.

The right kidney remained small with reduced perfusion, delayed peak uptake, and heterogeneous parenchymal uptake. The GFR was 62 mL/min. Excretion was reduced and delayed, with persistent pyelocaliceal and ureteral stasis. The renogram revealed a flattened peak, a plateaued excretory segment until diuretic administration, delayed T1/2, and increased residual activity. A non-obstructive response to furosemide was seen, but stasis persisted in the excretory pathways.

The functional distribution was 77% for the left kidney and 23% for the right kidney, with a total GFR of 288 mL/min. The findings suggested a hypoplastic right kidney with delayed excretion and a compensatory hypertrophic left kidney with ureterohydronephrosis and delayed excretion, improving post-diuretic.

The third scintigraphy, performed when the patient was 2 years and 3 months old, revealed an enlarged left kidney with regular contours, normal perfusion, and homogeneous parenchymal uptake. The GFR was 148 mL/min. Excretion was spontaneous but slow and reduced until diuretic administration. Persistent stasis was noted in the pyelocaliceal and ureteral systems, with a tortuous ureter. The renogram indicated a normal parenchymal peak at 3 min, an excretory plateau with a maximum at 18 min, and delayed T1/2 at 55 min with increased residual activity. Post-diuretic T1/2 normalized, but stasis persisted.

The right kidney remained hypoplastic, with reduced perfusion, heterogeneous parenchymal uptake, and a GFR of 52.9 mL/min. Excretion was slow and reduced, with pyelocaliceal stasis. The renogram showed a delayed and flattened peak, a plateaued excretory segment with a delayed descent, and increased residual activity. Post-diuretic T1/2 normalized, but mild intrarenal stasis persisted.

**Figure 6:**
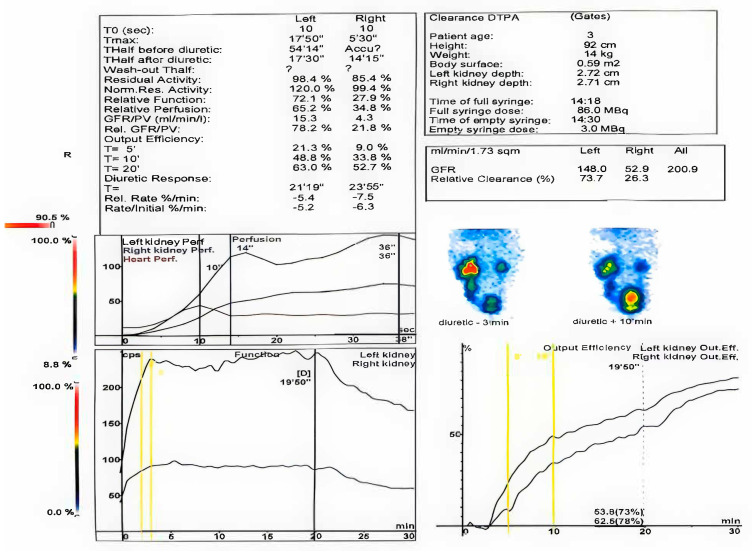
The third renal scintigraphy.

The differential functional contribution was 72.1% for the left kidney and 27.9% for the right kidney, with a total GFR of 200.9 mL/min. The findings confirmed a hypoplastic right kidney with reduced function and a hypertrophic left kidney with post-diuretic-responsive ureterohydronephrosis (Figure 6).

The fourth renal scintigraphy revealed a normal-sized left kidney with regular contours, normal perfusion, and homogeneous parenchymal uptake. Excretion was delayed, with persistent pyelocaliceal and ureteral stasis and a dilated renal pelvis. The renogram showed a flattened peak at 5 min, a progressively ascending excretory segment peaking at 20 min, and a non-obstructive post-diuretic response, although stasis persisted.

The right kidney remained small, with reduced perfusion and uptake and a GFR of 52.9 mL/min. Excretion was slow and reduced, with persistent intrarenal stasis. The renogram showed a slowly ascending peak at 16 min and a delayed excretory phase. Post-diuretic normalization was observed, with no residual stasis at the study’s end.

**Figure 7:**
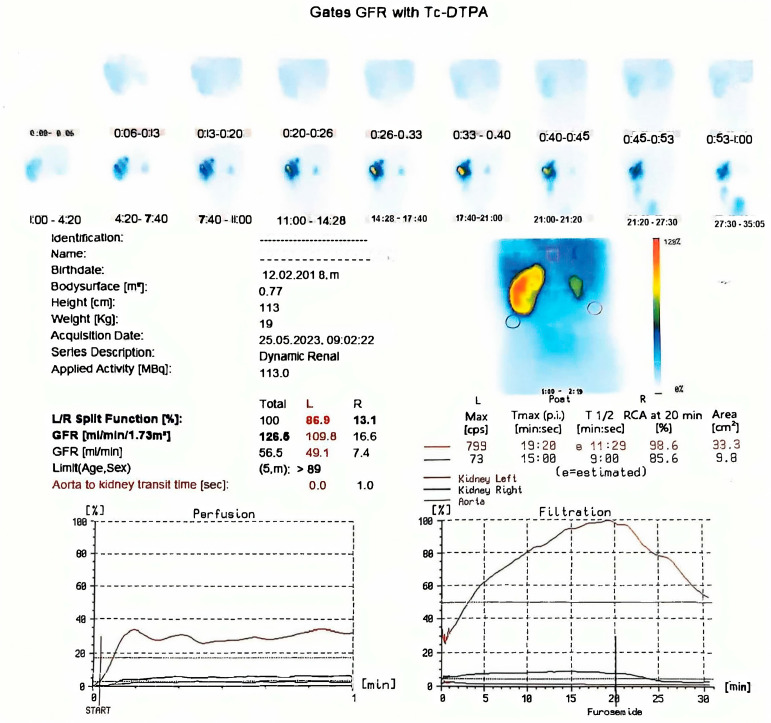
The fourth renal scintigraphy.

The differential function was 87% for the left kidney and 13% for the right kidney, with a total GFR of 126.5 mL/min. The conclusion was a hypoplastic right kidney with diminished function and a left kidney with ureterohydronephrosis and persistent stasis, despite responsiveness to diuretic (Figure 7).

**Figure 8:**
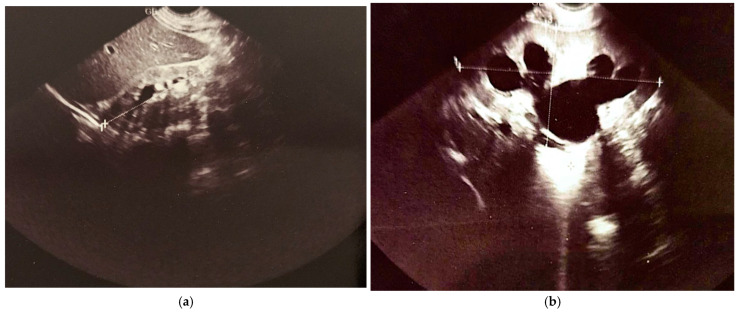

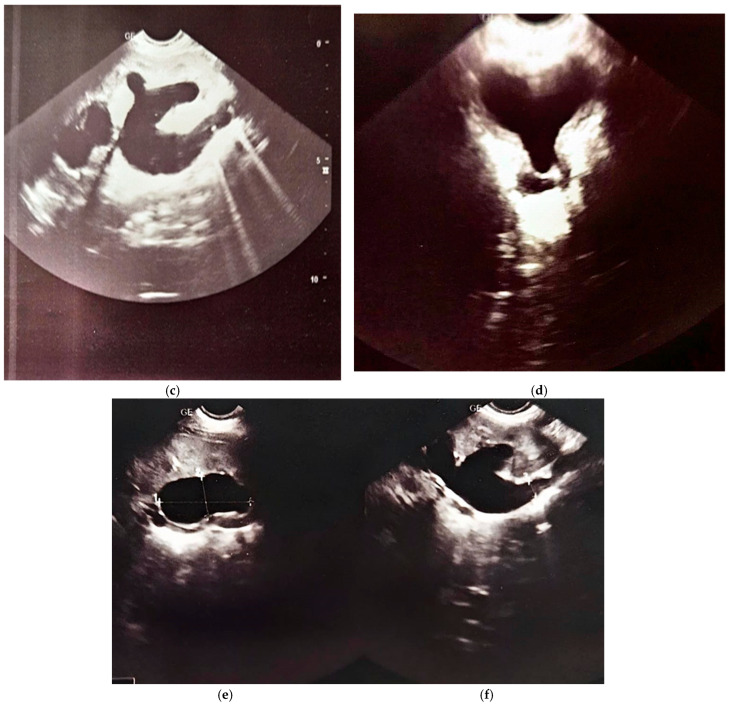
Renal ultrasound: (**a**) hypoplastic right kidney; (**b**) pelvicalyceal dilation of the left kidney; (**c**) dilated renal pelvis and ureter on the left kidney; (**d**) bladder with a modified (“funnel-shaped”) appearance; (**e**) pelvic dilation of the left kidney; (**f**) ureter dilated at the hilum of the left kidney.

The last abdominal ultrasound performed at 7 years of life revealed that the right kidney, located in the renal fossa, appeared hypoplastic. It maintained a regular shape and contour and was mobile with respiration. Its dimensions were 6.7 × 2.4 cm, with a parenchymal index (PI) of 9 mm. The pyelocaliceal system of the right kidney showed mild distension in the upper calyceal group, and no calculi were present. In contrast, the left kidney, also situated in the renal fossa, displayed a regular shape and contour with good respiratory mobility. It measured 10.4 × 4.8 cm, with a PI of 11 mm at the upper pole. Notably, the pyelocaliceal system of the left kidney was dilated; the renal pelvis and calyceal groups measured approximately 3.3 × 3 cm, and no calculi were observed. The left ureter was clearly visible at the renal hilum, measuring approximately 11 mm in diameter. The urinary bladder contained anechoic fluid and exhibited thickened walls. The left ureter could be visualized in its lower third, measuring about 8 mm in diameter, while the right ureter was seen at its entry into the bladder. The initial conclusions drawn from this ultrasound included grade III/IV hydronephrosis of the left kidney, a hypoplastic right kidney, and a status post complete repair of pyeloureteral duplication. The final conclusions further clarified that the right kidney demonstrated grade I hydronephrosis, the left kidney showed grade III/IV hydronephrosis, and there was evidence of a neurogenic bladder, all in the context of a previously completed repair of pyeloureteral duplication on the right side (Figure 8).

### 2.5. Long-Term Follow-Up

Over the following years, the patient’s condition required ongoing multidisciplinary care. Regular monitoring of renal function and imaging were conducted to evaluate hydronephrosis and ureteral patency. Recurrent UTIs were managed with both oral and intravenous antibiotics. Additional surgical interventions included the removal of ureteral stents and exploratory cystoscopy to address persistent bladder diverticula. At the age of five, the patient demonstrated clinical stabilization, with a serum creatinine of 46 μmol/L, an improved hydration status, and the absence of acute UTIs. However, imaging continued to show residual left hydronephrosis and a hypoplastic right kidney, underscoring the need for long-term surveillance and potential future interventions.

## 3. Discussion

This case illustrates the challenges encountered in managing severe congenital anomalies of the kidney and urinary tract (CAKUT), particularly when multiple complex abnormalities coexist. Early prenatal and postnatal detection of bilateral grade IV/V hydronephrosis with right pyeloureteral duplication, a left ureterocele, and a neurogenic bladder was pivotal in initiating prompt interventions. The initial decompression through bilateral ureterostomy and stenting led to significant clinical improvements, as demonstrated by a reduction in serum creatinine levels from 262 μmol/L at three days old to 28 μmol/L by ten days, thereby stabilizing renal function during the early neonatal period. However, despite these early gains, the persistent left hydronephrosis and right kidney hypoplasia underscore the critical need for long-term surveillance and individualized management strategies.

### 3.1. Diagnostic Approaches and Their Role in Management

The prenatal ultrasound detection of severe hydronephrosis enabled timely postnatal evaluations that confirmed the diagnosis and guided urgent intervention. Urinary ultrasound remains the gold standard for diagnosing and monitoring hydronephrosis [18,19,20,21,22,23], and in our patient, serial ultrasound studies consistently identified the extent of renal pelvis dilation and parenchymal thickness. Despite a generally benign course in neonatal hydronephrosis—with resolution rates strongly correlated with Society for Fetal Urology (SFU) grades [24]—associated anomalies such as ureterocele and duplex systems can portend persistent obstruction or renal dysfunction. In this case, the early insertion of bilateral ureterostomies and stenting was instrumental in relieving the obstruction, as supported by improved creatinine levels and the stabilization of the clinical status.

### 3.2. Surgical Intervention and Timing

While early intervention in CAKUT is known to preserve renal function, the timing of surgical procedures remains a delicate balance. In our case, despite initial decompression, definitive corrective surgery was postponed due to the patient’s failure to reach the requisite weight, resulting in an extended hospital stay [25,26]. This delay exemplifies the practical challenges in pediatric surgery, where patient growth and stability are critical determinants of operative timing. Subsequent interventions, including bilateral ureteral reimplantation, excision of the left ureterocele, and removal of a fibroepithelial polyp, were performed to address residual anatomical abnormalities. Although these procedures led to partial improvement, the persistent left hydronephrosis and right renal hypoplasia necessitate ongoing monitoring and future individualized interventions.

### 3.3. Recurrent Infections and Multidisciplinary Management

The clinical course was further complicated by multiple episodes of febrile urinary tract infections (UTIs) caused by multidrug-resistant organisms such as *Klebsiella pneumoniae*, *Escherichia coli*, and *Pseudomonas aeruginosa*. These recurrent infections, which often required escalation from oral to intravenous antibiotic therapy, significantly impacted both short-term outcomes and long-term renal function. The integration of medical, imaging, and surgical data highlights that, in patients with complex CAKUT, a multidisciplinary approach is essential. Collaborative care involving pediatric urologists, nephrologists, and radiologists enhances both diagnostic accuracy and therapeutic efficacy [12,13,14,27].

Nonetheless, careful follow-up is vital for detecting cases that progress to obstruction [22]. However, consensus on invasive diagnostics and surgical intervention for asymptomatic primary UPJHN remains challenging [18,28]. Tc-99m-MAG3 diuretic renal scans provide essential functional data to differentiate obstructed from non-obstructed kidneys and guide treatment decisions [26]. A 99mTc-Mag3 scan with furosemide washout is commonly used in moderate-to-severe cases [29,30], while ultrasound evaluation using SFU grades further aids in assessing severity [6]. The management of UPJ obstruction requires a tailored surgical strategy to relieve obstruction and preserve renal function [31]. Endoscopic ureteric stenting is emerging as a minimally invasive alternative to open surgery in children under 4, with reported success rates around 83.5% [31,32]. In cases where stenting fails, definitive surgery is indicated. Early bladder filling during VCUG is critical to avoid contrast obscuring the ureterocele [33,34,35]. The incidences of VUR in duplex systems and the benefits of early endoscopic decompression—reducing sepsis and renal damage—align with prior studies [36,37,38]. Functional assessments via renography further guide decisions on procedures such as partial nephrectomy or ureteral reimplantation [39,40,41].

Studies from Boston Children’s Hospital and others have reported low complication rates in mild antenatal hydronephrosis, with few cases requiring surgery [42,43]. Long-term investigations in CAKUT and alternative techniques like low-loop cutaneous ureterostomy and refluxing ureteral reimplantation underscore the need for standardized protocols and predictive models [44,45,46,47].

### 3.4. Clinical Implications

The implications of these findings are significant for clinical practice. Recognizing that contrast material can obscure critical anatomical details during VCUG emphasizes the need for early imaging during bladder filling. This approach enhances diagnostic accuracy and informs the surgical strategy, particularly in complex cases with duplex systems. Endoscopic decompression, as demonstrated, not only lowers the immediate risk of sepsis and renal damage from recurrent infections but also optimizes conditions for any future reconstructive surgery by reducing ureteral dilation. By tailoring the timing and type of intervention, whether opting for expectant management, partial nephrectomy, or ureteral reimplantation, clinicians can better preserve renal function and improve long-term outcomes. These strategies underscore the importance of a multidisciplinary approach, integrating imaging, functional assessment, and surgical planning to manage complex congenital anomalies effectively.

This child faces a significant risk of advancing to end-stage kidney disease (ESKD), along with all the complications associated with chronic kidney disease (CKD), making them a future candidate for dialysis or a renal transplant. It is crucial to highlight that this child experiences a notably impaired quality of life, as their renal function depends on the hypertrophied left kidney, which is also affected by hydronephrosis. Ongoing follow-up with a pediatrician, pediatric nephrologist, urologist, and pediatric surgeon is essential. Regular evaluations and hospitalizations are necessary to monitor renal function. A repeat renal scintigraphy is scheduled for this year to assess kidney function.

### 3.5. Evidence-Based Interventions in the Management of Complex CAKUT: Literature Review

The management of complex congenital anomalies of the kidney and urinary tract (CAKUT) requires a multidisciplinary approach. Early decompression through bilateral ureterostomies has been demonstrated to stabilize renal function by reducing obstruction-induced nephron loss, thereby preventing irreversible fibrosis and CKD progression. Studies have reported improvements in eGFR, even though residual hypoplasia may persist [16,48,49,50]. In contrast, delayed ureteral reimplantation is associated with ongoing vesicoureteral reflux, progressive renal scarring, and recurrent UTIs, ultimately increasing the risk of CKD and dialysis dependency [51,52,53]. Temporary diversions, such as ureterostomies, effectively provide decompression but carry a higher risk of multidrug-resistant infections compared with definitive repairs, which, despite requiring patient compliance, offer a more enduring correction [49,53,54,55]. Endoscopic interventions, though less invasive, tend to have higher recurrence rates than open surgical approaches, which have success rates exceeding 90% in the context of complex anatomical features, such as duplication or ureterocele with associated bladder polyps [16,25,56]. Effective management of neurogenic bladder through clean intermittent catheterization combined with anticholinergics significantly reduces UTI frequency—a critical factor for preserving renal function [53,55,56,57]. Although genetic testing was not performed in our patient, approximately 10–15% of CAKUT cases have been linked to monogenic mutations; thus, a genetic and multidisciplinary evaluation can enhance diagnostic precision and inform management strategies [48,58]. Finally, long-term surveillance is essential, as nearly 30% of CAKUT patients progress to CKD; persistent residual hydronephrosis and renal hypoplasia, as observed in our patient, underscore the need for ongoing nephrology and urology follow-up to monitor renal function and prevent further complications [48,49,50,57,59] (Table 2).

### 3.6. Limitation and Future Directions

Following the initial surgery (cutaneous ureterostomy), the patient experienced a 13-month wait before being scheduled for the subsequent procedure (ureteral reimplantation). This delay was attributed to anesthetic risks, a weight deficit, and recurrent urinary tract infections. Additionally, the scarcity of specialized centers for this procedure posed a challenge, as only one facility in our country was equipped to perform it.

Notwithstanding the advances in imaging and early intervention, certain limitations persist. The use of VCUG, while critical for assessing vesicoureteral reflux and ureterocele anatomy, is constrained by contrast-related obscuration, which may lead to underdiagnosis or mischaracterization of the abnormalities [31,32,33]. Similarly, variability in renal function assessments via renography can influence surgical decision-making [26]. Moreover, the inherent heterogeneity in patient presentations—as seen in this case—limits the generalizability of current management protocols. Future research should focus on refining imaging protocols, possibly by incorporating contrast-enhanced ultrasound or magnetic resonance urography, to overcome these limitations. Prospective studies are warranted to validate long-term outcomes of early decompression versus definitive reconstructive surgery and to develop standardized criteria for assessing upper pole functionality and determining the optimal timing for surgical interventions [42,43,44,45].

## 4. Conclusions

This case report highlights the considerable diagnostic and therapeutic challenges associated with managing severe bilateral congenital hydronephrosis complicated by additional urological anomalies, including pyeloureteral duplication, ureterocele, and neurogenic bladder. Early prenatal diagnosis allowed for timely initial intervention, significantly stabilizing renal function. However, the patient’s clinical course was marked by recurrent, severe urinary tract infections caused by multidrug-resistant pathogens, delayed surgical interventions due to anesthetic risks, weight deficits, and limited availability of specialized treatment centers.

The necessity of a multidisciplinary approach involving pediatric urologists, nephrologists, radiologists, and surgeons was underscored, significantly contributing to the effective management, diagnostic accuracy, and stabilization of renal function. Despite initial success with bilateral ureterostomy and subsequent reconstructive surgery, long-term complications such as residual left hydronephrosis, right kidney hypoplasia, and recurrent infections persisted, emphasizing the importance of ongoing surveillance and tailored individualized management.

Future directions must focus on refining diagnostic imaging techniques, exploring emerging technologies like contrast-enhanced ultrasound or magnetic resonance urography, and developing clear, standardized criteria for surgical decision-making. Prospective studies are needed to further validate outcomes associated with various surgical approaches and timing, aiming to enhance the precision of management strategies and improve long-term renal and overall health outcomes in children affected by complex congenital anomalies of the kidney and urinary tract (CAKUT).

## Figures and Tables

**Table 1:** Patient infections were identified and treated in the hospital.

Hospitalization Period	Patient’s Age	Identified Pathogens	Administered Antibiotic
26–29 November 2018	9 months	*Klebsiella pneumoniae*	Ceftriaxone (800 mg/day) + Gentamicin (50 mg/day)
15–24 December 2018	10 months	*Escherichia coli* (acute pyelonephritis)	Amikacin (128 mg/day)
23–28 February 2019	1 year	*Pseudomonas aeruginosa*, *Staphylococcus aureus*	Gentamicin (60 mg/day)
3 March 2019	1 year, 1 month	*Enterococcus*	Unspecified

**Table 2:** Evidence-based interventions in the management of complex CAKUT.

Intervention	Key Outcomes	Supporting Evidence	Relevance to Case	Citations
Early Decompression (Bilateral Ureterostomies)	- Stabilizes renal function short-term	Neonatal decompression prevents irreversible fibrosis and CKD progression in severe hydronephrosis [16,48].	Improved eGFR post-decompression (65 → 45 mL/min/1.73 m^2^) but residual hypoplasia.	[49,50]
	- Reduces obstruction-induced nephron loss			
Delayed Ureteral Reimplantation	- Higher CKD risk due to persistent VUR	Delayed repair linked to progressive scarring and dialysis dependency in adulthood [51,52].	Recurrent UTIs persisted post-reimplantation, necessitating further interventions.	[53]
	- Increased UTI recurrence			
Temporary vs. Definitive Diversions	- Temporary diversions (ureterostomies) carry infection risks	Vesicostomy provides decompression but requires meticulous care; augmentation cystoplasty introduces metabolic risks [54,55].	Recurrent MDR UTIs post-ureterostomy; reimplantation reduced VUR but not hydronephrosis.	[49,53]
	- Definitive repair requires compliance			
Endoscopic vs. Open Surgery	- Endoscopic methods are less invasive but have a higher recurrence	Endoscopic valve ablation resolves obstruction in 70–80% of neonates; open pyeloplasty achieves >90% success [16,25].	Open reimplantation chosen due to anatomical complexity (duplication, polyp).	[25,56]
	- Open surgery offers durable correction			
Neurogenic Bladder Management (CIC + Anticholinergics)	- Reduces UTI frequency by 50%	CIC improves bladder emptying and compliance, reducing UTI risk in neurogenic bladder [57].	Post-reimplantation CIC reduced UTIs from 6/year to 1–2/year.	[53,55,56]
	- Critical for graft survival post-transplant			
Genetic and Multidisciplinary Evaluation	- 10–15% of CAKUT linked to monogenic mutations	Genetic testing (e.g., *PAX2*, *HNF1B*) and multidisciplinary care improve diagnostics and outcomes [48,58].	Patient’s hypoplasia and dysplasia suggest genetic underpinnings; no testing reported.	[48,58].
	- CNVs contribute to 4–6% of cases			
Long-Term Surveillance	- 30% of CAKUT patients progress to CKD	CAKUT patients transitioning to adulthood require nephrology/urology follow-up [48,57].	Residual left hydronephrosis and right hypoplasia underscore need for ongoing care.	[49,50,59]
	- Lifelong monitoring for UTIs and renal function			

## Data Availability

Data are contained within the article.
